# The Role of Social Media in Knowledge, Perceptions, and Self-Reported Adherence Toward COVID-19 Prevention Guidelines: Cross-Sectional Study

**DOI:** 10.2196/44395

**Published:** 2024-02-16

**Authors:** Camryn Garrett, Shan Qiao, Xiaoming Li

**Affiliations:** 1 Department of Health Promotion, Education, and Behavior Arnold School of Public Health The University of South Carolina Columbia, SC United States

**Keywords:** COVID-19, digital media, social media, TikTok, Instagram, Twitter, Facebook, prevention guidelines

## Abstract

**Background:**

Throughout the COVID-19 pandemic, social media has served as a channel of communication, a venue for entertainment, and a mechanism for information dissemination.

**Objective:**

This study aims to assess the associations between social media use patterns; demographics; and knowledge, perceptions, and self-reported adherence toward COVID-19 prevention guidelines, due to growing and evolving social media use.

**Methods:**

Quota-sampled data were collected through a web-based survey of US adults through the Qualtrics platform, from March 15, 2022, to March 23, 2022, to assess covariates (eg, demographics, vaccination, and political affiliation), frequency of social media use, social media sources of COVID-19 information, as well as knowledge, perceptions, and self-reported adherence toward COVID-19 prevention guidelines. Three linear regression models were used for data analysis.

**Results:**

A total of 1043 participants responded to the survey, with an average age of 45.3 years, among which 49.61% (n=515) of participants were men, 66.79% (n=696) were White, 11.61% (n=121) were Black or African American, 13.15% (n=137) were Hispanic or Latino, 37.71% (n=382) were Democrat, 30.21% (n=306) were Republican, and 25% (n=260) were not vaccinated. After controlling for covariates, users of TikTok (β=–.29, 95% CI –0.58 to –0.004; *P*=.047) were associated with lower knowledge of COVID-19 guidelines, users of Instagram (β=–.40, 95% CI –0.68 to –0.12; *P*=.005) and Twitter (β=–.33, 95% CI –0.58 to –0.08; *P*=.01) were associated with perceiving guidelines as strict, and users of Facebook (β=–.23, 95% CI –0.42 to –0.043; *P*=.02) and TikTok (β=–.25, 95% CI –0.5 to -0.009; *P*=.04) were associated with lower adherence to the guidelines (*R*^2^ 0.06-0.23).

**Conclusions:**

These results allude to the complex interactions between online and physical environments. Future interventions should be tailored to subpopulations based on their demographics and social media site use. Efforts to mitigate misinformation and implement digital public health policy must account for the impact of the digital landscape on knowledge, perceptions, and level of adherence toward prevention guidelines for effective pandemic control.

## Introduction

In March 2020, the infectious disease SARS-CoV-2, more commonly known as COVID-19, was classified as a pandemic [1,2]. As the virus is transmitted through the respiratory systems of individuals in close contact, preventative measures include wearing a facial mask, social distancing, and receiving recommended COVID-19 vaccinations [3]. Over the course of the pandemic, prevention recommendations changed in response to emerging scientific evidence. Initially, a 14-day quarantine and isolation were recommended, which was then shortened to 10 days, and was once more shortened to 5 days [3]. As of March 2022, masks were still recommended in indoor spaces, COVID-19 vaccinations and boosters were widely available, and rapid self-testing was advised in response to exposure or symptom onset [3]. In the United States, as of November 2, 2022, there have been over 97 million confirmed cases and over 1 million total deaths due to COVID-19 [4]. Despite these prevention recommendations, case numbers continued to rise, necessitating research into prevention efforts.

In response to social distancing recommendations, many aspects of life shifted from physical to online environments. Adapting to this change, most US adults (ie, 90%) indicated that digital media was either essential or important for them throughout the pandemic [5]. Digital media encapsulates social media as the platforms that enable human connection in the online environment, with varying degrees of privacy [6]. On social media, individuals encounter and consume information, government announcements, and reactions from other users as they work, learn, connect, and are entertained online [7]. Popular social media sites include Facebook, Twitter, Instagram, Snapchat, TikTok, Pinterest, Reddit, and LinkedIn, among others. As of 2021, a total of 72% of adults in the United States report using at least 1 social media site, representing a 3% increase since 2018 [8]. When stratified by age, 84% of US adults aged 18-29 years indicate using at least 1 social media site [8]. Of those who use Facebook, Snapchat, and Instagram, a majority indicate visiting the platform at least once a day [9]. In considering news consumption on social media, when stratified by age, 42% of users aged 18-29 years indicate social media as their primary source of news [9].

With an increasing proportion of individuals active on social media, thereby encountering COVID-19 news and information online, there are concerns about information accuracy, where unsourced or false information that is widely distributed threatens the dissemination of scientifically accurate information [7,10]. The modalities of social media (eg, concise, organized content formats, and sharing capabilities) allow information to quickly trend as a result of high engagement. The visibility of trending content on social media is determined by engagement and is often based on sensationalism rather than factual accuracy [7]. Sensational misinformation risks reducing the visibility and reach of reputable information [7]. Due to the saturation of misinformation online, the United States is understood to be in a syndemic, denoting the interactions between the COVID-19 pandemic and the infodemic. Social media, therefore, has the capacity to serve both as a tool and a hindrance to health communication.

Despite motivations for use, social media users are subject to unintentionally overconsuming content related to COVID-19 due to the saturation of pandemic information online. Social media has been preliminarily found to negatively contribute to COVID-19 prevention guideline adherence [11]. Among US adults, 53.3% indicate that the amount of information on COVID-19 is overwhelming to the effect that 54.7% indicate that it has led to their avoidance of consuming information about COVID-19 [12]. Resembling emerging trends in the United States, a study in Turkey indicated that 34.4% of respondents follow COVID-19 guidelines less in the present than at the beginning of the pandemic [13]. Fluctuations in pandemic prevention perceptions and adherence over time can be expected, but negative trends, regardless of their cause, necessitate investigation and intervention to bolster commitment to prevention guidelines to limit further pandemic-related exposures [13]. Although a complicated mechanism with additionally probable explanations (eg, milder virus mutations, vaccination availability, mental health burdens, and pandemic fatigue), these downward patterns of adherence are thought to be partially explained by social media use (eg, misinformation and overconsumption). The effective dissemination of scientific, evidence-based health communication must be prioritized in stark opposition to skepticism and disbelief, as sustained by misinformation.

There exists a limited understanding of the associations between demographics and frequency of social media site use and engagement with pandemic prevention behaviors, despite the significant risks to public health. Therefore, there is a present and pressing need to address the field’s limited understanding of pandemic-related knowledge, perceptions, and adherence, as impacted online, to design effective health behavior and communication interventions. As the emerging literature demonstrates that content consumption impacts perceptions and, subsequently, health behaviors, the field of health communication must understand the compounding effects of the online environment on COVID-19 prevention efforts [7]. This study therefore aims to investigate the associations between the social media platforms from which individuals consume pandemic-related information as well as their frequency of use and their knowledge of, perceptions of, and adherence to COVID-19 prevention guidelines.

## Methods

### Survey Development and Data Collection

Preliminary development of the survey involved compiling constructs related to the topics of interest. Survey items were then drafted to measure participant knowledge, perceptions, and adherence toward COVID-19 prevention guidelines. The items were then reviewed by an expert to evaluate and ensure readability, applicability, and response options. The data were obtained using a web-based survey fielded using Qualtrics paid, opt-in distribution services. The data were collected from March 15, 2022, to March 23, 2022.

### Ethical Considerations

The University of South Carolina’s Institutional Review Board exempted the study (Pro00119512) from Human Research Subject Regulations based on its minimal risk to participants in providing web-based survey responses. Informed consent was obtained from all participants prior to survey completion. All participants were compensated for their time and efforts in completing the survey (ie, US $6).

### Sample

All adults in the United States were eligible for participation, given that they were 18 years or older at the time of survey response. Responses that were deemed low quality based on response speed, lack of variability in selection, or repetitive attempts were removed before analysis to ensure data quality. Qualtrics used quota sampling methods to ensure the collection of a sample proportionate to that of the United States by way of gender, age, income, race, ethnicity, and education level. The final sample size included 1043 viable responses.

### Measures

#### Demographics

Participant demographics collected included age, gender identity, race or ethnicity, education, employment, income, political affiliation, and COVID-19 vaccination status. Due to limited representation, the American Indian or Alaska Native and Native Hawaiian or Pacific Islander categories were collapsed into 1 category. Age, education, employment, and income were used as continuous variables in the regression models. Gender identity, race or ethnicity, political affiliation, and COVID-19 vaccination status were used as categorical variables in the regression models.

#### Frequency of Social Media Use

Participants’ frequency of any social media use was measured through the item: “About how often do you use social media sites?” Response options ranged from several times a day, once per day, a few times per week, once per week, less than once per week, to never.

#### Social Media Sources of COVID-19 Information

Participants were asked to check all that apply to the question, “Which of these social media sites have you used to get information about COVID-19?” with the possible response options of Facebook, Twitter, Instagram, Snapchat, Pinterest, TikTok, Reddit, LinkedIn, and another social media site. The social media sites available as response options were chosen due to their popularity and presentation of short-form, user-generated content. Although there exist additional social media platforms (eg, YouTube), those chosen to be included here have active engagement and content sharing capabilities. Demographic profiles of the included social media sites were not accounted for in participant sampling procedures, as it is assumed that user bases may have fluctuated during the pandemic. The selections of these sites were operationalized as categorical predictors in the regression models.

#### Knowledge of COVID-19 Guidelines

Set forth by the Centers for Disease Control and Prevention, as of March 2022, relevant COVID-19 guidelines were used in crafting 4 items to assess participant pandemic-related knowledge. The assessment evaluated respondents’ knowledge of calculating exposure date, the minimum length of isolation after an exposure or positive test, the percentage of alcohol in hand sanitizer required to kill COVID-19, and what a negative rapid test result indicates. Participants were asked to indicate what they believe the current, official recommendations to be, at the time of survey administration, rather than what they may prefer them to be. These 4 items were then compiled for a final score out of 100%. Knowledge scores of the COVID-19 prevention guidelines were used continuously in the regression models.

#### Perceptions of COVID-19 Guidelines

Participants were asked to indicate the degree to which they perceived COVID-19 prevention guidelines to be relaxed or strict. The terminology “strict” was operationalized through concurrent dimensions that encapsulate participant responses to legal and scientific guidelines as well as enforcement. As perceptions of COVID-19 guidelines were assessed after the knowledge assessment, the guidelines were not explicitly defined but rather assumed to encapsulate mask-wearing, gathering size limitations, hygiene measures, as well as quarantine and isolation timelines. This ordering provided participants with context as to what the term “guidelines” referred to. Participants were asked: “Do you consider the current COVID-19 guidelines as:” with the response options ranging from too strict, a little too strict, about right, a little too relaxed, to too relaxed.

#### Adherence to COVID-19 Guidelines

Adherence to COVID-19 guidelines was evaluated by asking participants if they generally follow the official COVID-19 prevention guidelines, with the available response options of strongly, sometimes, rarely, and never follow the guidelines. This item provided an average, typical measure of self-reported participant adherence to COVID-19 guidelines, broadly. Given the state of the pandemic, this item was reliant upon participant understanding of guidelines in the organizations and institutions to which they belong (ie, schools and workplaces).

### Statistical Analysis

All statistical analyses were conducted using the statistical analysis software, SAS (version 9.4; SAS Institute). Descriptive analyses were conducted for key predictors. All data were screened for outliers, missing data, and normality. As all data used in this study was collected through discrete response options, excluding age, their distributions were considered to assess the presence of outliers. This was done by considering the frequency of responses within available options through histograms and box plots, as applicable. Those categories that were lower in response volume were collapsed (eg, race or ethnicity response of American Indian or Alaska Native and Native Hawaiian or Pacific Islander) or excluded from the analysis before modeling (eg, gender identity response option of nonbinary). Data quality was ensured as Qualtrics excluded participants who did not complete the survey in a single session, who were not continuously and carefully responding, who missed embedded attention checks, or who completed the survey in less than a third or more than 3 times the median time it took other participants to complete the survey. Due to the use of these features, respondents who did not complete the survey were not tracked. No systematic patterns of missing data within the data collected, or between variables, were observed. There is limited item nonresponse. Bivariate associations were assessed through ANOVA and Pearson correlation tests, as appropriate. Three generalized linear regressions, using a maximum likelihood estimation procedure, were conducted, independently, to explore associations between social media use and demographics and knowledge, perceptions, and self-reported adherence toward prevention guidelines, respectively. Although the 3 outcomes of knowledge, perceptions, and self-reported adherence were run independently, their theoretically dependent nature led us to consider implementing a correction (ie, Bonferroni), but as it resulted in a minimal impact on our findings, the traditional α level of .05 was here used to evaluate our findings.

## Results

### Overview

Of the 1043 participants, the median age of participants was 45.3 years (Table 1). The distribution of the gender identity of the participants was split approximately equally between men (515/1032, 49.9%) and women (513/1032, 49.71%), with few participants indicating being nonbinary or transgender. The race or ethnicity of participants was primarily White (696/1042, 66.79%), followed by Latino or Hispanic (137/1042, 13.15%) and Black or African American (121/1042, 11.61%). A quarter (253/1042, 24.28%) of participants held a bachelor’s degree and approximately a quarter (269/1042, 25.82%) of participants indicated earning US $50,000-US $79,999 annually. Finally, almost half (498/1040, 47.88%) of the participants had received a full vaccination series and booster against COVID-19.

**Table 1:** Demographic characteristics of study participants (N=1043).

Variables			Values, n (%)
Age (years;1 participant’s data are missing), mean (SD)			45.3 (16.94)
**Gender** **(11 participants’ data are missing)**			
	Men	515 (49.9)	
	Women	513 (49.71)	
	Nonbinary or other	4 (0.39)	
**Race or ethnicity (check all that apply; 1 participant’s data are missing)**			
	Black or African American	121 (11.61)	
	Latino or Hispanic	137 (13.15)	
	American Indian or Alaska Native and Native Hawaiian or Pacific Islander	22 (2.11)	
	White	696 (66.79)	
	Other	66 (6.33)	
**Education** **(1 participant’s data are missing)**			
	Less than high school degree	25 (2.4)	
	High school graduate or equivalent	248 (23.8)	
	Some college but no degree	248 (23.8)	
	Associate degree	123 (11.8)	
	Bachelor’s degree	253 (24.28)	
	Master’s degree	112 (10.75)	
	Doctoral or professional degree (JD, MD, or PhD)	33 (3.17)	
**Employment status over the last 3 months** **(6 participant’s data are missing)**			
	Working full-time	499 (48.12)	
	Working part-time	132 (12.73)	
	Unemployed and looking for work	74 (7.14)	
	Homemaker or stay-at-home parent	70 (6.75)	
	Student	35 (3.38)	
	Retired	200 (19.29)	
	Other	27 (2.6)	
**Previous year income (US $; 1 participant’s data are missing)**			
	Less than 10,000	56 (5.37)	
	10,000-19,999	58 (5.57)	
	20,000-29,999	96 (9.21)	
	30,000-39,999	87 (8.35)	
	40,000-49,999	70 (6.72)	
	50,000-59,000	117 (11.23)	
	60,000-69,999	70 (6.72)	
	70,000-79,999	82 (7.87)	
	80,000-89,999	47 (4.51)	
	90,000-99,999	51 (4.89)	
	100,000-149,999	215 (20.63)	
	150,000 or more	93 (8.93)	
**Political affiliation** **(30 participants’ data are missing)**			
	Republican	306 (30.21)	
	Democrat	382 (37.71)	
	Independent	325 (32.08)	
**COVID-19 vaccination status (3 participant’s data are missing)**			
	No	260 (25)	
	Yes, but no booster	282 (27.12)	
	Yes, including booster	498 (47.88)	

### Social Media Site Use

Participants reported using, generally or for any reason, the social media sites Facebook (835/1042, 80.13%), Twitter (396/1042, 38%), Instagram (586/1042, 56.24%), Snapchat (329/1042, 31.57%), Pinterest (320/1042, 30.71%), TikTok (401/1042, 38.48%), Reddit (208/1042, 19.96%), LinkedIn (254/1042, 24.38%), or another social media site (69/1042, 6.62%). Further, participants reported accessing COVID-19 information using the social media sites Facebook (604/1042, 57.97%), Twitter (220/1042, 21.11%), Instagram (258/1042, 24.76%), Snapchat (85/1042, 8.16%), Pinterest (59/1042, 5.66%), TikTok (129/1042, 12.38%), Reddit (84/1042, 8.06%), LinkedIn (72/1042, 6.91%), and another social media site (42/1042, 4.03%).

Table 2 presents the results of the bivariate analyses. Pearson correlations suggest that the demographic variables of age, education, and income were correlated with the prevention mitigation outcomes of guideline knowledge, perceptions, and self-reported adherence. The ANOVA suggests that political affiliation was correlated with all 3 outcomes while gender, race or ethnicity, and COVID-19 vaccination status were correlated with prevention guideline perceptions and self-reported adherence. Social media sites used to consume COVID-19 news were correlated with self-reported adherence. Employment and regularity of social media use were not correlated with the outcomes of interest.

**Table 2:** Bivariate analysis results.

Variable		Outcomes		
		Knowledge	Perceptions	Self-reported adherence
**Age**				
	*r*	0.09	–0.14	0.08
	*P* value	.006	<.001	.01
**Education**				
	*r*	0.11	0.001	0.11
	*P* value	<.001	.97	<.001
**Employment**				
	*r*	0.02	–0.03	0.04
	*P* value	.48	.27	.21
**Income**				
	*r*	0.15	–0.08	0.04
	*P* value	<.001	.007	.17
**Gender**				
	ANOVA (*F*)	0.38	6.43	5.27
	*P* value	.54	.01	.02
**Race or ethnicity**				
	ANOVA (*F*)	2.36	12.66	3.85
	*P* value	.051	<.001	.004
**Political affiliation**				
	ANOVA (*F*)	6.23	94.13	49.87
	*P* value	.002	<.001	<.001
**COVID-19 vaccination status**				
	ANOVA (*F*)	2.7	23.88	69.85
	*P* value	.07	<.001	<.001
**Site for COVID-19 news**				
	ANOVA (*F*)	2.07	1.64	2.89
	*P* value	.07	.15	.01
**Regularity of social media use**				
	ANOVA (*F*)	0.53	1.21	1.23
	*P* value	.75	.30	.29

### Knowledge of COVID-19 Guidelines

Indicating the level of knowledge related to COVID-19 prevention guidelines, the possible scores participants could receive included 100% (n=14, 1.4%), 75% (n=112, 10.9%), 50% (n=429, 41.7%), 25% (n=368, 35.7%), or 0% (n=107, 10.4%) correct. Model 1 (Table 3) suggests that income, Democratic political affiliation, and use of the social media platform TikTok were associated with COVID-19 prevention guideline knowledge. Specifically, as income (β=.03, 95% CI 0.005-0.05; *P*=.02) increased, it was found to be associated with a higher level of knowledge of COVID-19 guidelines. Democratic political affiliation (β=–.21, 95% CI –0.37 to –0.057; *P*=.008) was found to be negatively associated with guideline knowledge. Using TikTok as a source of COVID-19 information (β=–.29, 95% CI –0.58 to –0.004; *P*=.047) was associated with a lower level of knowledge. This model explained 6% of the variance in knowledge of COVID-19 guidelines.

**Table 3:** Regression results for knowledge, perceptions, and self-reported adherence.

Independent variables (reference)			Model 1: knowledge^a^					Model 2: perceptions^a^					Model 3: self-reported adherence^a^		
			β (95% CI)		*P* value		β (95% CI)			*P* value		β (95% CI)			*P* value
Age			.002 (–0.003 to 0.007)		.51		–.007 (–0.01 to –0.002)			.007^b^		.004 (–0.0004 to 0.008)			.07
**Gender** **(men)**															
	Women	.026 (–0.1 to 0.15)		.69		.16 (0.02 to 0.3)			.02^b^		.15 (0.04 to 0.26)			.008^b^	
**Race or ethnicity** **(White)**															
	Black or African American	–.048 (–0.26 to 0.16)		.66		.14 (–0.09 to 0.36)			.24		.21 (0.03 to 0.39)			.02^b^	
	Hispanic or Latino	–.079 (–0.27 to 0.11)		.41		.28 (0.08 to 0.49)			.007^b^		.27 (0.11 to 0.43)			.001^b^	
	American Indian or Alaska Native and Native Hawaiian or Pacific Islander	.072 (–0.45 to 0.6)		.79		.92 (0.35 to 1.49)			.002^b^		.32 (–0.13 to 0.77)			.16	
	Other	–.09 (–0.35 to 0.17)		.49		.07 (–0.21 to 0.34)			.63		.14 (–0.07 to 0.36)			.19	
Education level			.03 (–0.014 to 0.079)		.17		–.015 (–0.065 to 0.036)			.56		.001 (–0.039 to 0.04)			.95
Employment			.029 (–0.008 to 0.066)		.13		–.001 (–0.04 to 0.038)			.95		.005 (–0.027 to 0.036)			.78
Income			.03 (0.005 to 0.05)		.02^b^		–.03 (–0.053 to –0.005)			.02^b^		–.015 (–0.03 to 0.004)			.13
**Political affiliation** **(independent)**															
	Republican	–.12 (–0.28 to 0.04)		.15		–.5 (–0.67 to –0.33)			<.001^b^		–.23 (–0.37 to –0.09)			.001^b^	
	Democrat	–.21 (–0.37 to –0.057)		.008^b^		.34 (0.17 to 0.5)			<.001^b^		.17 (–0.04 to 0.31)			.01^b^	
**COVID-19 vaccination status** **(yes, but no booster)**															
	No	.00 (–0.17 to 0.17)		.99		–.22 (–0.4 to –0.04)			.02^b^		–.22 (–0.36 to –0.07)			.003^b^	
	Yes, including booster	.02 (–0.13 to 0.18)		.78		.31 (0.15 to 0.48)			<.001^b^		.32 (0.19 to 0.45)			<.001^b^	
**Site for COVID-19 news** **(Reddit)**															
	Facebook	–.086 (–0.31 to 0.14)		.45		–.23 (–0.47 to 0.009)			.06		–.23 (–0.42 to –0.043)			.02^b^	
	Instagram	–.026 (–0.28 to 0.23)		.84		–.40 (–0.68 to –0.12)			.005^b^		–.22 (–0.44 to 0.0026)			.05	
	Snapchat	.21 (–0.26 to 0.68)		.38		–.17 (–0.66 to 0.31)			.49		–.33 (–0.71 to 0.057)			.10	
	TikTok	–.29 (–0.58 to –0.004)		.047^b^		–.29 (–0.6 to 0.016)			.06		–.25 (–0.5 to –0.009)			.04^b^	
	Twitter	.015 (–0.22 to 0.25)		.90		–.33 (–0.58 to –0.08)			.01^b^		–.08 (–0.28 to 0.12)			.43	
**Regularity of social media use** **(less than once per week)**															
	Several times per day	.27 (–0.18 to 0.71)		.24		–.22 (–0.71 to 0.27)			.37		–.24 (–0.62 to 0.14)			.22	
	Once per day	.16 (–0.32 to 0.63)		.52		–.12 (–0.63 to 0.4)			.66		–.23 (–0.63 to 0.18)			.27	
	A few times per week	.2 (–0.29 to 0.69)		.43		–.03 (–0.57 to 0.5)			.91		–.16 (–0.58 to 0.27)			.47	
	Once per week	–.55 (–1.33 to 0.22)		.16		–.23 (–1.07 to 0.62)			.60		–.03 (–0.7 to 0.63)			.92	

^a^*R*^2^ values of models 1-3 are 0.06 (knowledge), 0.23 (perceptions), and 0.19 (self-reported adherence) respectively.

^b^*P* values indicate statistical significance at the α=.05 level.

### Perceptions of COVID-19 Guidelines

Model 2 (Table 3) suggests that age, gender, Hispanic or Latino populations, American Indian or Alaska Native populations, income, political affiliation, COVID-19 vaccination status, and the use of the social media sites Instagram and Twitter were associated with perceptions of COVID-19 prevention guidelines. As age (β=–.007, 95% CI –0.01 to –0.002; *P*=.007) increased, it was found to be associated with a perception of the guidelines as strict. Women (β=.16, 95% CI 0.02-0.3; *P*=.02) were associated with perceiving the guidelines as relaxed. Hispanic or Latino (β=.28, 95% CI 0.08-0.49; *P*=.007) and American Indian or Alaska Native and Native Hawaiian or Pacific Islander (β=.92, 95% CI 0.35-1.49; *P*=.002) populations were found to be associated with perceiving the guidelines as relaxed. As income (β=–.03, 95% CI –.05 to –.005; *P*=.02) increases, it was found to be associated with stricter perceptions of the guidelines. Republican political affiliation (β=–.5, 95% CI –0.67 to –0.33; *P*<.001) was found to be associated with perceiving the guidelines as strict, while Democratic political affiliation (β=.34, 95% CI 0.17-0.5; *P*<.001) was found to be associated with perceiving them as relaxed. Receiving the full vaccination series and booster (β=.31, 95% CI 0.15-0.48; *P*<.001) was found to be associated with perceiving the guidelines as relaxed, while receiving no COVID-19 vaccinations (β=–.22, 95% CI –0.4 to –0.04; *P*=.02) was associated with perceiving them as strict. Instagram (β=–.4, 95% CI –0.68 to –0.12; *P*=.005) and Twitter (β=–.33, 95% CI –0.58 to –0.08; *P*=.01) were found to be associated with stricter perceptions of the COVID-19 prevention guidelines. This model explained 23% of the variance in perceptions of COVID-19 guidelines.

### Adherence to COVID-19 Guidelines

As related to self-reported COVID-19 guideline adherence, model 3 (Table 3) suggests that women, Black or African American populations, Hispanic or Latino populations, political affiliation, COVID-19 vaccination status, and the use of Facebook and TikTok were associated with adherence to the COVID-19 prevention guidelines. Women (β=.15, 95% CI 0.04-0.26; *P*=.008) were found to be positively associated with adherence to the COVID-19 prevention guidelines. Black or African American (β=.21, 95% CI 0.03-0.39; *P*=.02) and Hispanic or Latino (β=.27, 95% CI 0.11-0.43; *P*=.001) populations were found to be positively associated with adherence to the guidelines. Republican political affiliation (β=–.23, 95% CI –0.37 to –0.09; *P*=.001) was negatively associated with adherence to prevention guidelines, while Democratic political affiliation (β=.17, 95% CI –0.04 to 0.31; *P*=.01) was positively associated with adherence. Receiving the full vaccination series and booster (β=.32, 95% CI 0.19-0.45; *P*<.001) was positively associated with adherence to the COVID-19 prevention guidelines, while receiving no COVID-19 vaccinations (β=–.22, 95% CI –0.36 to –0.07; *P*=.003) was negatively associated with adherence. Facebook (β=–.23, 95% CI –0.42 to –0.043; *P*=.02) and TikTok (β=–.25, 95% CI –0.5 to –0.009; *P*=.04) were found to be negatively associated with self-reported adherence to COVID-19 prevention guidelines. This model explained 19% of the variance in adherence to COVID-19 guidelines.

## Discussion

### Principal Findings

This study suggests that knowledge, perceptions, and self-reported adherence toward COVID-19 prevention guidelines differ by demographics and social media site use. Notably, marginalized populations (eg, older adults, women, and racial or ethnic minority individuals) were found to perceive the COVID-19 prevention guidelines as relaxed, in addition to their positive association with adherence. Political affiliation and COVID-19 vaccination status mirror assumptions about perceptions and adherence, where those identifying as Republican and reporting no vaccination were associated with perceiving the guidelines as too strict and adhering to a lesser degree, respectively. The popular social media sites TikTok, Instagram, Facebook, and Twitter were found to negatively impact pandemic prevention efforts as they were differentially associated with lower levels of knowledge, perceiving guidelines as strict, and lower self-reported adherence. The findings of this work, while demonstrating complicated interactions between guideline knowledge, perceptions, and adherence, serve to inform tailored public health interventions (ie, on the basis of demographic subgroups and social media site use), platform policies (eg, misinformation prevention), and digital public health policy more broadly.

### Demographics and Knowledge, Perceptions, and Adherence Toward Guidelines

When considering the associations between the demographic correlates of income, age, and gender with knowledge, perceptions, and adherence toward prevention guidelines, the findings suggest a complex pandemic landscape. Whereas education and employment were not associated with guideline knowledge, it can be assumed that income reflects a layer of privilege afforded to those of higher income throughout the pandemic. In the case of this study, income may be acting as a proxy for pandemic privilege rather than solely socioeconomic status. Pandemic privilege can be understood here as the role of income in altering the pandemic environment, where those with additional resources are more likely to have access to prevention methods (eg, working from home, personal protective equipment, vaccination appointment flexibility, transportation, residential privilege, limited disruptions to services and care, and financial buffer for burdens of lost employment and wages) [14,15]. Despite possessing increased knowledge of the guidelines, perceptions of the prevention guidelines as strict reflect privileged protections afforded through increased income. Concordant with the existing literature, among older adults, a higher level of adherence to prevention guidelines, despite perceptions of them as strict, is likely due to the higher risk of severe illness from COVID-19 associated with increased age [16,17]. Gendered differences in perceptions of the guidelines as relaxed with a higher level of adherence reflect disproportionate pandemic burdens experienced by women (eg, occupational exposure, incidence, and post–COVID-19 condition [long COVID]).

The present findings are in accordance with the existing literature that demonstrates the impact of political affiliation on knowledge, perceptions, and adherence toward prevention guidelines. Partisan differences in perceptions of COVID-19 guidelines have been theorized to be explained by differential risk perceptions as influenced by news sources and media consumption [18-21]. Republican political affiliation has been found to be aligned with a preference for reducing the imposition of guidelines, while Democratic political affiliation is aligned with a preference for maintaining guidelines [22]. In accordance with the literature, political affiliation may play a decisive role in impacting knowledge-seeking and comprehension, perceptions, and adherence toward prevention guidelines. Health communication efforts may bolster prevention efforts through the characteristics inherent to partisan politics (eg, collectivism, inequity perceptions, perceived risk, skepticism, and media influence) and their influence on health behaviors [22-24]. The emerging literature attests that although political affiliation may demonstrate explanatory differences in pandemic prevention outcomes, there is a call for public health efforts that extend beyond interventions targeted based on political affiliation, implementing bipartisan efforts that also further consider demographics and individual differences influencing the operationalization of information from news and social media sites in the interest of COVID-19 prevention [18,23].

### Social Media Sites and Knowledge, Perceptions, and Adherence Toward Guidelines

The use of the social media sites TikTok, Instagram, Twitter, and Facebook was found to be associated with lower knowledge, stricter perceptions, and lesser adherence toward COVID-19 prevention guidelines. Despite operating under distinct algorithms, all 4 platforms share commonalities in their functions for photo, video, audio, and text sharing, as well as social networking structures. A reliance on user-generated content creates difficulty in regulating the presence and spread of misinformation on social media. All 4 sites implemented, to various degrees, efforts to mitigate misinformation through informational banners on videos discussing the pandemic with off-site links to additional information. Despite these soft moderation efforts to address misinformation by TikTok, Instagram, Twitter, and Facebook, all have been found to contribute to the dissemination of misinformation [25-28]. Therefore, there is a need for improved mechanisms on these social media sites to limit the spread of misinformation due to its negative impacts on COVID-19 prevention guideline knowledge, perceptions, and adherence in the physical environment.

One key consideration of this study is the discrepancy between the demographic profiles of the included social media sites and the study sample. The user base of TikTok (ie, 48% users aged 18-29 years, 22% users aged 30-49 years, 14% users aged 50-64 years, and 4% users aged 65 years and older), Twitter (ie, 42% users aged 18-29 years, 27% users aged 30-49 years, 18% users aged 50-64 years, and 7% users aged 65 years and older), and Instagram (ie, 71% users aged 18-29 years, 48% users aged 30-49 years, 29% users aged 50-64 years, and 13% users aged 65 years and older) tends to be younger than that of Facebook (ie, 70% users aged 18-29 years, 77% users aged 30-49 years, 73% users aged 50-64 years, and 50% users aged 65 years and older) [8]. Although the average age of the study sample is older, it aligns with profiles of users of a similar age range who are active online (ie, 22% users on TikTok, 27% users on Twitter, 48% users on Instagram, and 77% users on Facebook) [8]. Although social media sites have unique demographic user profiles, it is necessary to consider that all individuals are able to access their platforms. Understanding the scope of a platform’s typical and atypical users is necessary to systematically address misinformation online, where those who do not align with the average user experience an assumedly differential interaction with the platform and its content.

### Public Health Implications

This research is uniquely situated within the COVID-19 pandemic and serves to inform tailored public health interventions, social media platform strategies, and policies. The key implications of this research include addressing knowledge gaps in the literature regarding the impact of social media use and demographic characteristics on COVID-19 prevention guideline knowledge, perceptions, and adherence. Public health interventions should be tailored to relevant platforms to address the impacts of social media sites on prevention guideline knowledge, perceptions, and adherence. Additionally, interventions targeting demographic subgroups may be operationalized on social media platforms with a user base that aligns with the target subgroup (eg, age, income, and political affiliation). In this context, platform functionality should be considered when designing interventions, regulations, and misinformation mitigation policies to alleviate the negative impacts of social media use on COVID-19 prevention efforts. Finally, these findings are necessary to be operationalized within public health interventions to tailor interventions to increase pandemic-related knowledge while enhancing supportive perceptions of the guidelines, aiming to increase and maintain sufficient adherence among subpopulations to mitigate the effects of the pandemic.

### Strengths, Limitations, and Future Studies

This study has the strengths of using a country-wide, quota-based sample to investigate emerging trends during the pandemic as related to knowledge of, perceptions of, and adherence to COVID-19 prevention guidelines. Although there is likely some inherent difference in those who are online and able to participate in the survey as compared with those who are not, this concern may be mitigated in the context of this work, as it centers those active in the online environment. With the goal of identifying the role of social media on the target population, the exclusion of those not online is warranted. The findings should be cautiously interpreted and generalized as selection bias may affect the representativeness of the sample. When interpreting the study’s findings, low statistical significance does not imply the absence of a certain phenomenon. One limitation that could persist, as the results are reliant on a self-report measure of prevention guidelines adherence, is participants’ ability to approximate habits (eg, wearing a mask and using a social media site). A key limitation of this study is the discrepancy between the demographics of the study sample and the demographic profiles of the users of the various social media sites included. Finally, as a cross-sectional study, where some potential but key confounders may not have been included, there is the inability to obtain causal inference. Further, work accounting for the interrelations between factors should be conducted to provide a comprehensive assessment of confounders [22]. Future work should consider focusing on the validation of measures to assess knowledge, perceptions, and adherence. Additional research would benefit from an expanded survey considering a variety of potential, influential factors (eg, health literacy and location). Longitudinal explorations of the influence of social media use, knowledge levels, and declining perceptions should be prioritized in efforts to examine their impacts on prevention guideline adherence over time. Future directions for health communication should prioritize implementing programmatic interventions on social media platforms to address misinformation and information oversaturation in a manner that optimizes each platform’s social networking functions, algorithms, and user base.
